# Arthroscopic treatment of synovial chondromatosis of the ankle, an uncommon location: a case report

**DOI:** 10.1097/RC9.0000000000001131

**Published:** 2026-10-21

**Authors:** Andi Praja Wira Yudha Luthfi, Audie Djiady, Carmen Claude Margo, Frederika Frederika, Mitchel Mitchel, Erica Kholinne

**Affiliations:** a Department of Orthopedics and Traumatology, Bhayangkara Tk.I Pusdokkes Polri Hospital, Jakarta, Indonesia; b Department of Orthopedic and Traumatology, Gatam Institute, Eka Hospital, Tangerang, Indonesia; c Faculty of Medicine and Health Science, Atma Jaya Catholic University of Indonesia, Jakarta, Indonesia; d Department of Surgery, Faculty of Medicine, Universitas Trisakti, Jakarta, Indonesia

**Keywords:** ankle joint, arthroscopy, case report, loose bodies, synovial chondromatosis

## Abstract

**Introduction and importance:**

Synovial chondromatosis (SC) is a rare benign metaplastic disorder characterized by the formation of intra-articular cartilaginous loose bodies. Although it commonly affects large joints, ankle involvement is uncommon and may lead to delayed diagnosis and progressive joint damage. Arthroscopic management of ankle SC has been infrequently reported.

**Case presentation:**

A 54-year-old active man presented with a 1-year history of progressive left ankle pain, crepitus, and restricted range of motion. Radiographs, computed tomography, and magnetic resonance imaging demonstrated multiple intra-articular loose bodies and an anterior tibial spur. The patient underwent arthroscopic loose-body removal, complete anterior synovectomy, and anterior tibial spur resection through standard anteromedial and anterolateral portals. Histopathological examination confirmed synovial chondromatosis. Early weight-bearing was initiated on postoperative day 1. At three-month follow-up, the patient achieved complete pain relief, full range of motion, and no radiographic evidence of recurrence. At six-month follow-up, he remained free of complaints. At 9-month follow-up, the patient remained asymptomatic, with no clinical evidence of recurrence.

**Clinical discussion:**

Complete removal of loose bodies and synovectomy are essential to minimize recurrence. Arthroscopic treatment offers excellent joint visualization, minimal soft tissue disruption, and rapid functional recovery in appropriately selected cases.

**Conclusion:**

This case highlights that SC should be considered in patients with chronic ankle pain and mechanical symptoms. Arthroscopic management can provide excellent short-term clinical outcomes while minimizing surgical morbidity.

## Introduction

Synovial chondromatosis (SC) is a rare benign metaplastic disorder of the synovium characterized by the formation of cartilaginous nodules within the joint, although its exact etiology remains unknown^[^1^]^. It most commonly affects large joints such as the knee and hip, whereas foot and ankle involvement is uncommon^[^2^]^. The condition typically occurs in adults between the third and fifth decades of life and presents with pain, swelling, stiffness, and crepitus^[^3,4^]^. Although generally histologically benign, untreated disease may result in progressive cartilage degeneration, irreversible joint damage, and functional impairment. Management is primarily surgical, aiming to relieve symptoms, restore joint function, prevent further joint damage, and reduce recurrence. Treatment options include open arthrotomy and arthroscopic excision^[^1,2^]^. While arthroscopy is widely accepted for large joints because of its minimally invasive nature and favorable recovery, evidence for ankle SC remains limited to case reports and small case series, with no consensus regarding the optimal surgical approach, extent of synovectomy, or postoperative rehabilitation^[^5^]^. This report describes the arthroscopic management of ankle SC to further contribute to the limited literature on this uncommon condition.

HIGHLIGHTS

- Synovial chondromatosis of the ankle is a rare cause of chronic mechanical ankle pain.
- Magnetic resonance imaging and computed tomography facilitate accurate diagnosis and precise surgical planning.
- Arthroscopic loose body removal with synovectomy resulted in an excellent functional recovery.
- Early arthroscopic treatment may help alleviate symptoms.

In this report, we present a rare case of SC of the ankle joint, successfully treated with arthroscopic excision and synovectomy, highlighting the diagnostic considerations, surgical technique, and short-term clinical outcome. This case report has been presented in line with the SCARE checklist^[^6^]^.

## Case presentation

An active 54-year-old man presented to our hospital with a 1-year history of chronic left ankle pain. The pain (VAS score = 6) developed gradually and progressively worsened, particularly during weight-bearing and daily activities. During this period, the patient managed his symptoms at home with nonsteroidal anti-inflammatory drugs without seeking medical care. The patient denied any history of trauma, smoking, infection, allergies, or prior ankle surgery and had no known chronic illnesses or relevant comorbidities.

On physical examination, the ankle showed no erythema, warmth, or swelling. Palpable crepitus was elicited during ankle motion, accompanied by a restricted range of motion compared with the contralateral side. No joint instability or neurovascular deficit was identified.

Laboratory investigations, including complete blood count and inflammatory markers, were within normal limits, effectively excluding inflammatory or infectious etiologies. Plain radiographs of the left ankle revealed multiple well-defined intra-articular loose bodies and an associated anterior tibial spur (Figs 1A and B). Subsequent computed tomography (CT) scan and magnetic resonance imaging (MRI) confirmed the presence of multiple loose bodies within the ankle joint, predominantly involving the anterior and lateral compartments, without evidence of aggressive features or malignancy (Figs 1C–F). The differential diagnosis included anterior ankle impingement, osteoarthritis with loose-body formation, and synovial chondrosarcoma. Anterior ankle impingement was considered because of the anterior tibial spur and restricted range of motion, while osteoarthritis was considered because of the presence of intra-articular loose bodies. However, CT and MRI demonstrated multiple well-defined loose bodies without aggressive imaging features, making SC more likely.

**Figure 1.**
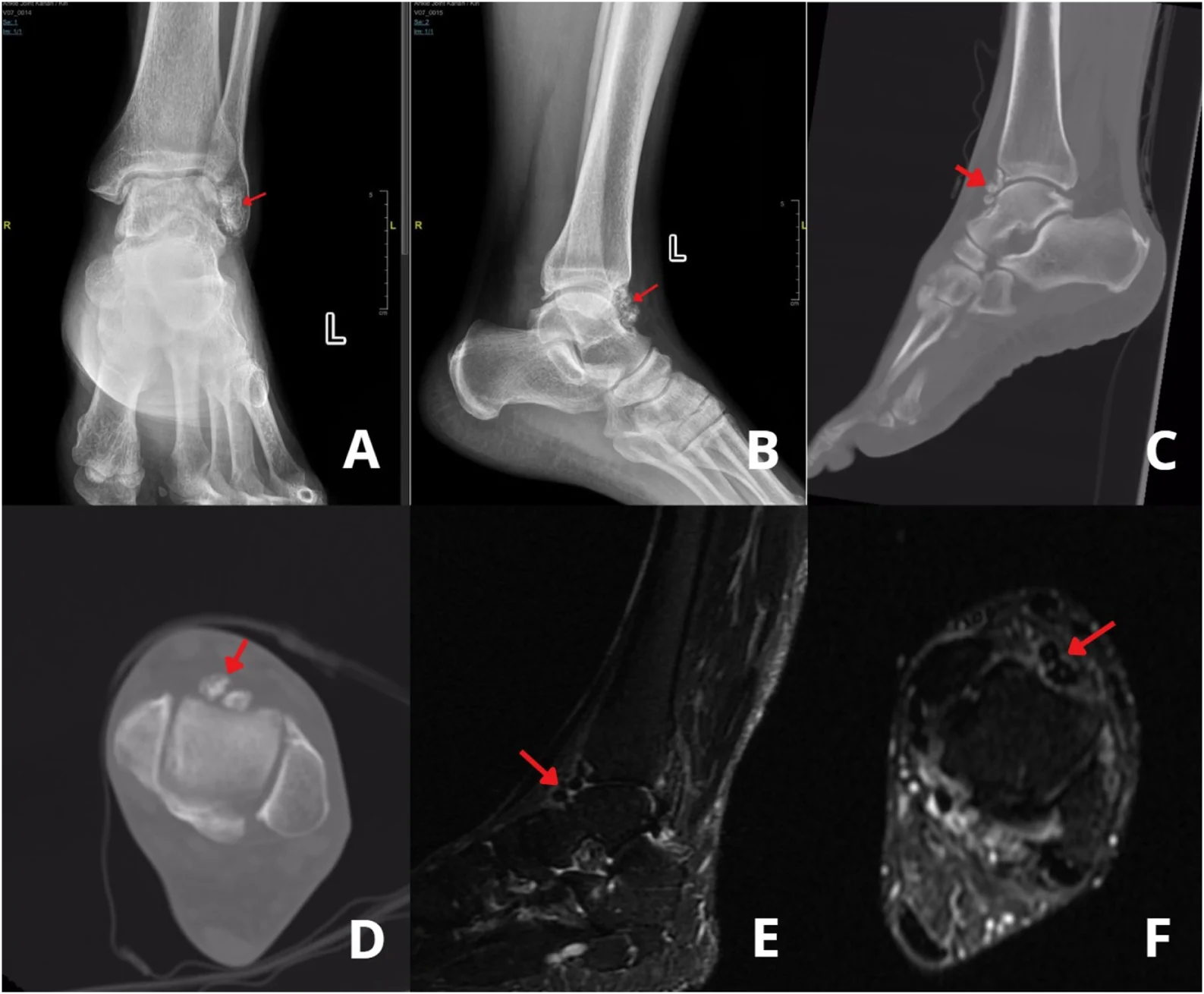
Plain radiograph of the left ankle in (A) AP view and (B) lateral view showing multiple intra-articular loose bodies (red arrow) and an anterior tibial spur. Computed tomography in (C) sagittal and (D) axial planes, and T2-weighted MRI in (E) sagittal and (F) axial planes of the left ankle, showing the presence of multiple intra-articular loose bodies (red arrow).

Given the persistent symptoms and mechanical limitations, the patient underwent ankle arthroscopy under spinal anesthesia using standard anteromedial and anterolateral portals. Arthroscopy revealed multiple cartilaginous loose bodies in the anterior and lateral gutters with hypertrophic synovium (Figs 2A,B). Complete loose-body removal, synovectomy, and anterior tibial spur resection were performed (Figs 2C,D). Histopathological examination of the excised loose bodies confirmed SC with no evidence of malignant transformation (Figs 3A,B).

**Figure 2.**
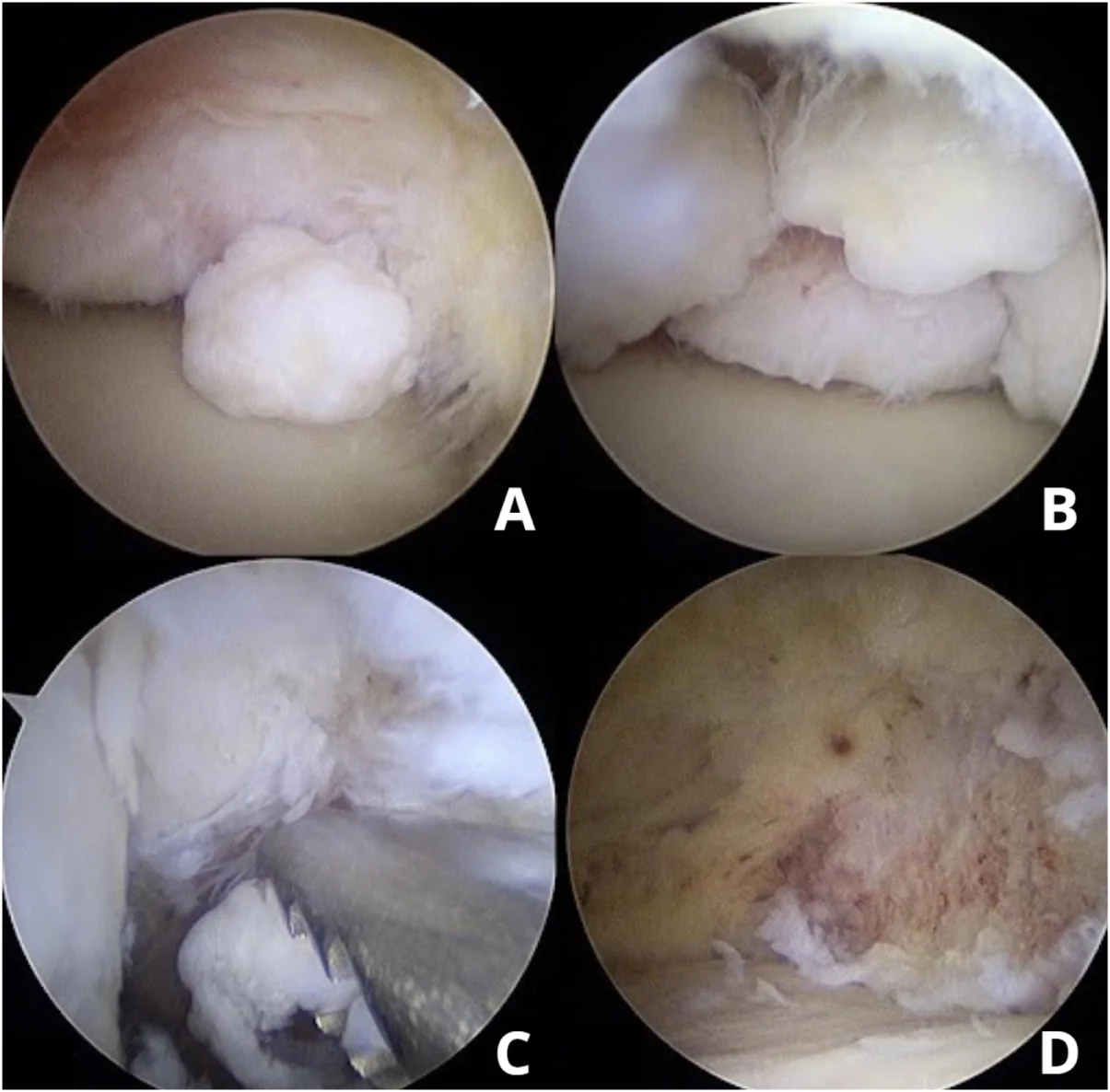
Intraoperative arthroscopic findings: (A, B) Multiple cartilaginous loose bodies; (C) Arthroscopic removal of loose bodies; (D) Arthroscopic view following complete loose-body removal and anterior tibial spur resection.

**Figure 3.**
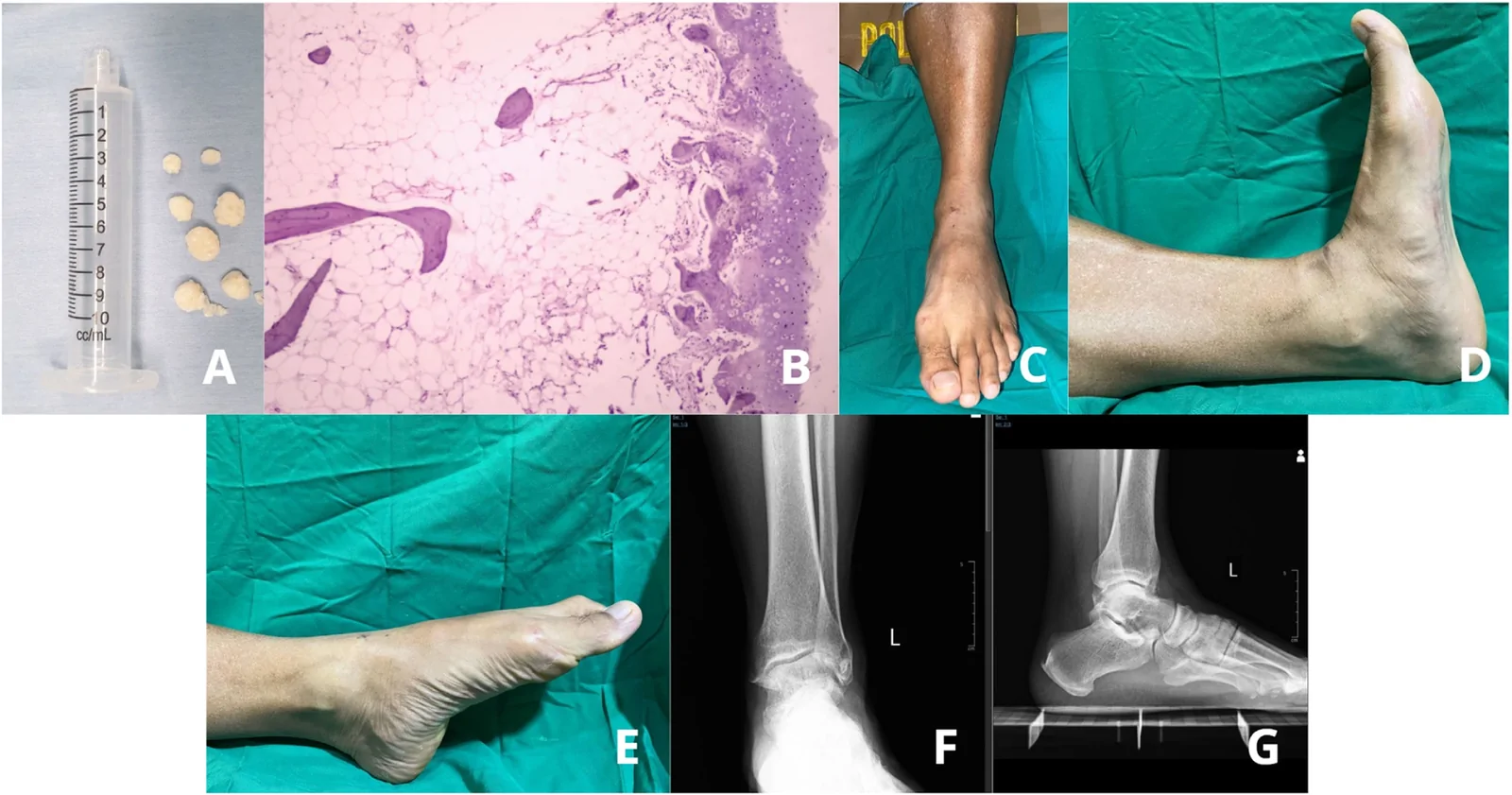
(A) Gross appearance of the excised intra-articular loose bodies. (B) Histopathological examination reveals cartilaginous nodules (hematoxylin and eosin staining, 100×). (C–E) Clinical appearance of the left ankle at 3 months postoperatively, demonstrating restored range of motion. (F, G) Follow-up radiograph of the left ankle at 3 months postoperatively shows no residual or recurrent loose bodies.

Postoperatively, the patient was allowed weight-bearing as tolerated starting on postoperative day 1, with early range-of-motion exercises encouraged. The postoperative course was uneventful. At the 3-month follow-up, the patient reported complete resolution of ankle pain (VAS = 0) and had regained full, painless range of motion (Figs 3C–E). He had returned to normal daily activities without limitation. Follow-up radiographs demonstrated no residual or recurrent loose bodies within the ankle joint (Figs 3F,G). At the 6-month follow-up, the patient remained free of any complaints. At the 9-month follow-up, the patient remained asymptomatic, with no clinical evidence of recurrence.

## Discussion

SC is a rare benign metaplastic disorder characterized by cartilaginous proliferation within the synovium, leading to the formation of intra-articular loose bodies^[^7^]^. Although the precise etiology remains unclear, several factors have been implicated, including embryonic remnants, genetic predisposition, infection, and trauma. Regardless of the initiating mechanism, ongoing synovial metaplasia results in progressive mechanical irritation of the joint, which may ultimately lead to cartilage degeneration and secondary osteoarthritic changes if left untreated^[^8^]^.

Milgram classified SC into three stages based on disease progression. Stage I is characterized by active synovial disease without loose bodies; stage II represents transitional disease with both active synovitis and loose bodies; and stage III consists of multiple loose bodies without active synovial proliferation^[^5^]^. Accurate staging is clinically relevant, as it guides surgical strategy. In stage II disease, as observed in our patient based on imaging findings and intraoperative observations, both loose body removal and synovectomy are recommended to address ongoing synovial activity and reduce the risk of recurrence.

Clinically, SC presents with joint pain aggravated by activity, restricted motion, swelling, and mechanical symptoms such as locking^[^9^]^. In the ankle, these manifestations are often nonspecific and may mimic anterior ankle impingement, degenerative arthritis, or inflammatory arthropathies, resulting in delayed diagnosis^[^10,11^]^. Untreated disease may lead to persistent joint irritation, cartilage degeneration, irreversible joint damage, and, rarely, malignant transformation into chondrosarcoma^[^10^]^. Therefore, early diagnosis and appropriate surgical intervention are essential to preserve joint function^[^12^]^.

Management of ankle SC depends on patient age, symptom severity, and disease stage. Complete synovectomy with removal of all loose bodies is recommended to minimize recurrence^[^13^]^. Both open arthrotomy and arthroscopy are effective treatment options. Arthroscopy is increasingly preferred because it provides superior visualization, less soft tissue injury, faster rehabilitation, and enables treatment of associated intra-articular pathology^[^14^]^. However, it may be less suitable for large loose bodies or extensive synovial involvement^[^15^]^. Open arthrotomy is generally reserved for large or multiple loose bodies, extensive disease, or incomplete arthroscopic excision, and may offer lower recurrence rates when complete synovectomy is achieved^[^14–16^]^. In contrast, arthroscopic management, while minimally invasive, has reported recurrence rates of approximately 7–23%, mainly due to incomplete lesion removal^[^16,17^]^.

A combined anterior–posterior arthroscopic approach has been suggested to facilitate complete removal of loose bodies when extensive disease is present^[^18^]^. However, in selected cases with disease predominantly involving the anterior compartment, an anterior arthroscopic approach may be sufficient. In our case, the use of standard anteromedial and anterolateral portals provided adequate visualization of the anterior and lateral gutters, allowing complete excision of loose bodies and thorough synovectomy without the need for additional posterior portals^[^13^]^. Despite the absence of posterior involvement on both preoperative imaging and intraoperative evaluation, the possibility of occult posterior synovial disease or future posterior loose body formation cannot be completely ruled out. The decision to use anterior portals alone was therefore guided by the predominantly anterior and lateral distribution of the lesions.

Compared with open arthrotomy, arthroscopic management of ankle SC offers several distinct advantages, including superior joint visualization, avoidance of extensive soft tissue dissection, lower postoperative morbidity, no requirement for prolonged cast immobilization, and faster functional rehabilitation, all of which contribute to quicker return to daily activities^[^19,20^]^. However, arthroscopy has notable limitations in the treatment of SC, particularly in cases with extensive or posteriorly located disease, where restricted visualization and instrument access may result in incomplete loose-body removal and residual synovial disease, thereby increasing the risk of recurrence^[^5,19^]^. The choice between arthroscopic and open surgery is therefore guided by several disease-related factors. The size and number of loose bodies are important considerations, as arthroscopy is generally adequate for numerous small fragments, but loose bodies exceeding approximately 2 cm may be difficult to remove intact through standard portals and often require open excision^[^2^]^. The extent of synovial proliferation is also relevant, since diffuse or bulky synovial involvement may compromise complete arthroscopic resection^[^2,5^]^. In addition, the specific joint affected plays a role, as smaller or more constrained joints, such as the ankle, offer less working space than larger joints, which can further limit instrument maneuverability and completeness of excision^[^2,21^]^. In such cases, open arthrotomy or a combined anterior–posterior arthroscopic approach may provide more extensive synovectomy and potentially reduce recurrence, although these approaches are associated with greater surgical morbidity and longer recovery^[^5^]^. In our case, the lesion consisted of multiple but subcentimeter loose bodies without extensive synovial proliferation and was predominantly localized to the anterior and lateral compartments, allowing complete excision through standard anterior portals.

This case highlights that SC should be considered in patients with chronic ankle pain, mechanical symptoms, and radiographic loose bodies. Prompt recognition and appropriate imaging are essential to prevent progressive joint damage. Arthroscopic loose-body removal with synovectomy can provide excellent functional outcomes and early mobilization and may minimize recurrence risk when complete excision is achieved.

This report describes a rare case of ankle synovial chondromatosis, including comprehensive imaging, histopathological confirmation, and successful arthroscopic treatment. However, the single-case design and limited follow-up duration restrict the assessment of long-term recurrence and generalizability. In addition, functional outcomes were primarily assessed through clinical descriptions and VAS pain scores, as validated patient-reported outcome measures were not collected. Further studies with larger cohorts, longer follow-up, and validated patient-reported outcome measures are warranted.

## Conclusion

Arthroscopic management provides a minimally invasive approach with excellent visualization, low morbidity, and rapid functional recovery. This case emphasizes the importance of considering SC in patients with chronic ankle pain and mechanical symptoms. Appropriate arthroscopic removal of loose bodies, along with synovectomy, may provide symptomatic relief and help preserve joint function. However, this single case cannot determine long-term outcomes or the risk of recurrence.

## Data Availability

Not applicable.
